# Insights into cationic ordering in Re-based double perovskite oxides

**DOI:** 10.1038/srep19746

**Published:** 2016-01-25

**Authors:** Tae-Won Lim, Sung-Dae Kim, Kil-Dong Sung, Young-Mok Rhyim, Hyungjeen Jeen, Jondo Yun, Kwang-Ho Kim, Ki-Myung Song, Seongsu Lee, Sung-Yoon Chung, Minseok Choi, Si-Young Choi

**Affiliations:** 1 Department of Nano Science and Engineering, Kyungnam University, Changwon 631-701, Korea; 2 Materials Modeling and Characterization Department, Korea Institute of Materials Science, Changwon 642-831, Korea; 3 Department of Physics, Pusan National University, Busan 609-735, Korea; 4 School of Materials Science and Engineering, Pusan National University, Busan 609-735, Korea; 5 Neutron Science Division, Korea Atomic Energy Research Institute, Daejeon 305-353, Korea; 6 Graduate School of EEWS, Korea Advanced Institute of Science and Technology, 291 Daehak-ro, Yuseong-gu, Daejeon 34141, Korea

## Abstract

Cationic ordering in Sr_2_FeReO_6_ (SFRO) and Sr_2_CrReO_6_ (SCRO) is investigated using magnetic property measurement, atomic-scale imaging, and first-principles calculations. We find that the nature of cationic ordering strongly depends on the host oxides, although they have the same crystal symmetry and chemical formula. Firstly, adding Re is effective to enhance the cationic ordering in SFRO, but makes it worse in SCRO. Secondly, the microscopic structure of antisite (AS) defects, associated with the level of cationic ordering, is also distinguishable; the AS defects in SFRO are clustered in the form of an antiphase-boundary-like feature, while they are randomly scattered in SCRO. Interestingly, we observe that the clustered AS defects deteriorate the ferromagnetism more than the scattered defects. Our findings elevate the importance of the AS defect configuration as well as the amount of defects in terms of magnetic property.

Double perovskite oxides have been extensively studied due to their half-metallic ferromagnetism, tunneling magneto-resistance, Curie temperature well above 300 K, and significant spin-orbit interactions and correlated electron behavior that can be utilized in nonvolatile logic devices^1^,^2^,^3^. Their crystal structure consists of a regular arrangement with the general formula A_2_BB′O_6_ or AA′B_2_O_6_. In practice, the B-cations of A_2_BB′O_6_ prefer an ordered pattern with positions fitting the rock-salt structure, whereas most of AA′B_2_O_6_ exhibits the layered A-cation ordering^4^. A fully ordered arrangement of cations in double perovskite oxides leads to maximized net magnetization. However, the arrangement can be disturbed, resulting in the formation of AS defects (e.g., B-on-B′ or vice versa in A_2_BB′O_6_), which correlates with deteriorated magnetic properties^5^,^6^,^7^. In A_2_BB′O_6_, for instance, AS defects can be defined as the percentage of B cations exchanged with B′ cations and vice versa, implying that a complete disorder is 50% defects. Therefore, many efforts have been made to quantify the level of cationic ordering to obtain good magnetic property with suppression of the AS defect formation. Conventionally, AS defects can be examined by diffraction methods since specific Bragg peaks such as (111), (113), and (331) appear due to the cationic ordering at the B and B′ superlattice^8^. Similarly, Mossbauer spectroscopy^9^ and NMR^10^,^11^ can be utilized to quantify the defects by investigating the amount of misplaced magnetic ions.

Here, we investigate SFRO and SCRO, which are prototypical magnetic materials (space group of *I4/m*). In the two oxides, the B-cation sublattice is associated with ferromagnetically arranged Fe^+3^ (3*d*^5^, *S* = 5/2) or Cr^+3^ (3*d*^3^, *S* = 3/2), and the Re^+5^ (5*d*^2^, *S* = 1) sublattice is antiferromagnetically coupled with the B-cation sublattice. If the level of B/B′ cationic ordering becomes lower, AS defects form such as Fe-on-Re (Fe_Re_) and Re-on-Fe (Re_Fe_) in SFRO; and Cr-on-Re (Cr_Re_) and Re-on-Cr (Re_Cr_) in SCRO, respectively, depending on the growth environment. Their formation reduces the magnetization of the oxides via strong antiferromagnetic coupling of defects and the host atoms. A previous study^12^ also reported that lowering the defect content increases the magnetic saturation and decreases the coercive magnetic field in SFRO. However, in the present study, we find that the magnetic properties are not simply dependent on the quantity of AS defects. For example, SFRO sample having 17.340% of AS defects decreases the magnetization value by ~50%. In contrast, SCRO sample with 17.378% of AS defects shows only ~10% decrease in the magnetization. This points that the magnetization is affected by the other factors besides the quantity of AS defects.

Our aim is to provide deep insight into the relationship between the AS defects and the magnetic properties in Re-based double perovskite oxides. A variety of SFRO and SCRO samples were prepared and characterized in terms of microstructure and magnetic properties by varying the amount of excessive Re, because the Re source is generally volatile during the sample preparation and thus can influence cationic ordering. Adding Re thus affords control over the concentration of AS defects. We performed microscopic analysis of the concentration and configuration of AS defects using x-ray diffraction, atomic-scale imaging, and first-principles calculations. The magnetic properties associated with AS defects are examined using magnetic hysteresis-loops measurement.

## Materials and Methods

### Materials and Characterization

SFRO and SCRO powders were prepared via conventional solid state reaction and spark plasma sintering (SPS; Sumitomo Coal Mining SPS-1050). SrCO_3_, Fe_2_O_3_ (Cr_2_O_3_), and ReO_3_ powders were mixed by ball-milling and pressed into pellets for SFRO (SCRO) precursors. AS defects can be controlled by synthesis conditions^5^, such as temperature, time, and pressure. In addition, the growth of stoichiometric Re-based double perovskites, especially SFRO, is straightforward due to the preference of Re-deficiency. In this regards, excess Re has been thus considered to control the quantity of AS defects^13^ The previous study also confirms that adding Re atoms helps to increase the cationic ordering and effectively improve the magnetic property of SFRO^13^. To this end, excess x-Re (x = 0, 5, 10, 15 mol%) were added to systematically change the AS defects. The pellets were calcined at 1000 °C for 10 hr in Ar atmosphere with heating and cooling rates of 7 °C/min, and sintered by SPS at 1150 °C for 10 min in Ar atmosphere of 65 MPa pressure. All sintered samples were polished to a thickness of ~1.0 mm, and were examined by x-ray diffraction (XRD; Rigaku D/Max 2500). Microstructures of AS defects were investigated by using high-angle annular dark-field scanning-transmission electron microscopy (HAADF-STEM; JEOL 2100F). Atomic arrangements are visualized via high angle scattered electrons, which reflects atomic number (*Z*). We used an image filter for enhancing the image quality of the STEM images. We used the radial difference filter module of the HREM-Filters built by HREM Research Inc. (high frequency maximum: 0.5, smooth edge: 0.3). For magnetic properties samples were cut with similar shape and size, and measured via vibrating sample magnetometer (VSM) option in a physical property measurement system (PPMS).

### First-principles calculations

The calculations were performed using the projector augmented-wave method^14^ and the Perdew-Burke-Ernzerhof (PBE)-GGA exchange-correlation functional^15^ with a Hubbard-*U* correction (GGA+*U*) as implemented in the VASP code^16^. The electronic wave functions were described using a planewave basis set with an energy cutoff of 400 eV. A rotationally invariant +*U* method^17^ was applied to the Fe 3*d* (*U*_eff_ = 4.0 eV), Cr 3*d* (*U*_eff_ = 3.0 eV), and Re 5*d* (*U*_eff_ = 1.4 eV) orbitals. For a few cases, calculations using the Heyd-Scuseria-Ernzerhof (HSE06) hybrid functional^18^,^19^, providing good physical descriptions of oxides^20^,^21^, were conducted to complement the GGA+*U* results. Spin polarization was considered in all the calculations. The calculated lattice parameter and magnetic moment of SFRO and SCRO using the GGA+*U* are listed in Table S1. The HSE values and experimental data are also included for comparison. The GGA+*U* and HSE06 provide similar spin-magnetic moment of each atom in the oxides, and the calculated values are acceptably close to experimental data, although the experimental values are scattered.

The calculations for antisite defects in SFRO and SCRO were performed using 320-atom supercells. The wavefunctions were expanded in a plane-wave basis set with an energy cutoff of 400 eV, and integrations over the Brillouin zone were carried out using the 2×2×2 *k*-point mesh. The atomic coordinates were relaxed until the force acting on each atom was reduced to less than 0.02 eV/Å.

## Results and Discussion

**Figure 1:**
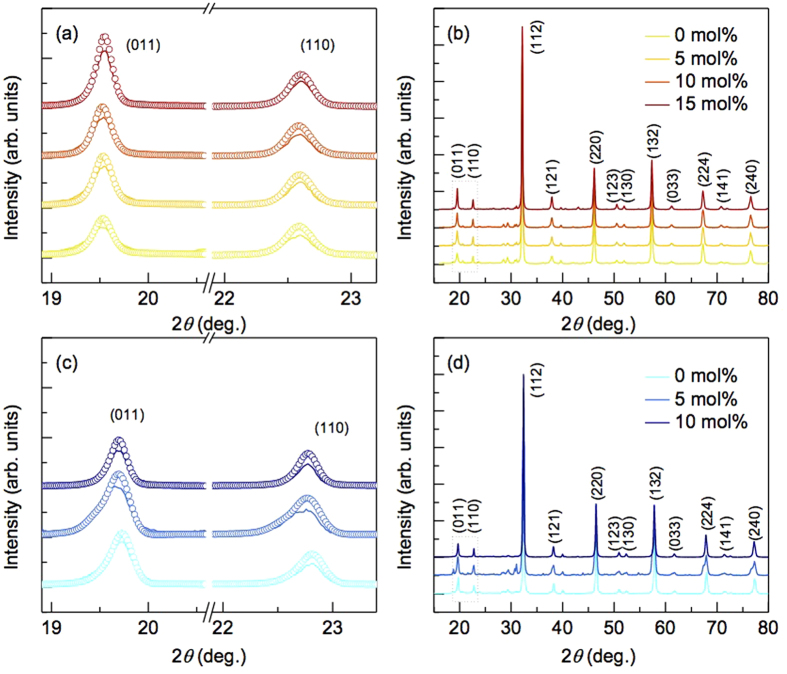
X-ray diffraction patterns for (**a,b**) SFRO-xRe (0 mol% ≤ x ≤ 15 mol%) and (**c,d**) SCRO-xRe (0 mol% ≤ x ≤ 10 mol%). The measured (011) and (110) peaks (solid lines) with refined results (open circles) for SFRO-xRe and SCRO-xRe are presented in (a,c), respectively.

Figure 1 shows XRD patterns of SFRO-xRe (0 ≤ x ≤ 15 mol%) and SCRO-xRe (0 ≤ x ≤ 10 mol%) whose main peaks are well matched to the crystal structure with the space group *I4/m*. Note that the unknown impurities are ignored in this study due to their minor effects on magnetic and microstructural properties with respect to the amount of excess Re. To address the correlation between the amount of excess Re and the cationic ordering, the peaks of SFRO-xRe and SCRO-xRe were refined by the Rietveld method using Fullproof program, and the results are listed in Table 1. Evaluating the ratio of the (110) and (011) peak intensity (*I*_110_/*I*_011_), a simple way to quantify the concentration of AS defects, also support the calculated the level of cationic ordering^22^. The results indicate that as amount of excess Re increases, the AS defect concentration decreases in SFRO by 10.4%, but slightly increases in SCRO by 0.9%. Consequently, adding excess Re produces stoichiometric and cation-ordered SFRO by suppressing the formation of AS defects, while it does not improve the quality of SCRO. Our fist-principles calculations suggest that the discrepancy is attributed to the thermodynamic stability of SFRO and SCRO under Re-excess growth conditions. The calculated chemical-potential diagram shows that SCRO occupies a ~25% smaller region compared with SFRO, and the areas of both the SFRO and SCRO regions are narrow (Figure S1). This means that growth of stoichiometric and fully cation-ordered samples is not straightforward, especially for SCRO. We experimentally confirm that excess 15 mol% Re gives rise to stoichiometric SFRO samples with a defect concentration of 10.512%, while it leads to nonstoichiometry in SCRO with major impurity phases (not shown). These findings indicate that the conventional understanding, excess Re induces enhancement of sample quality, is not robust.

**Figure 2:**
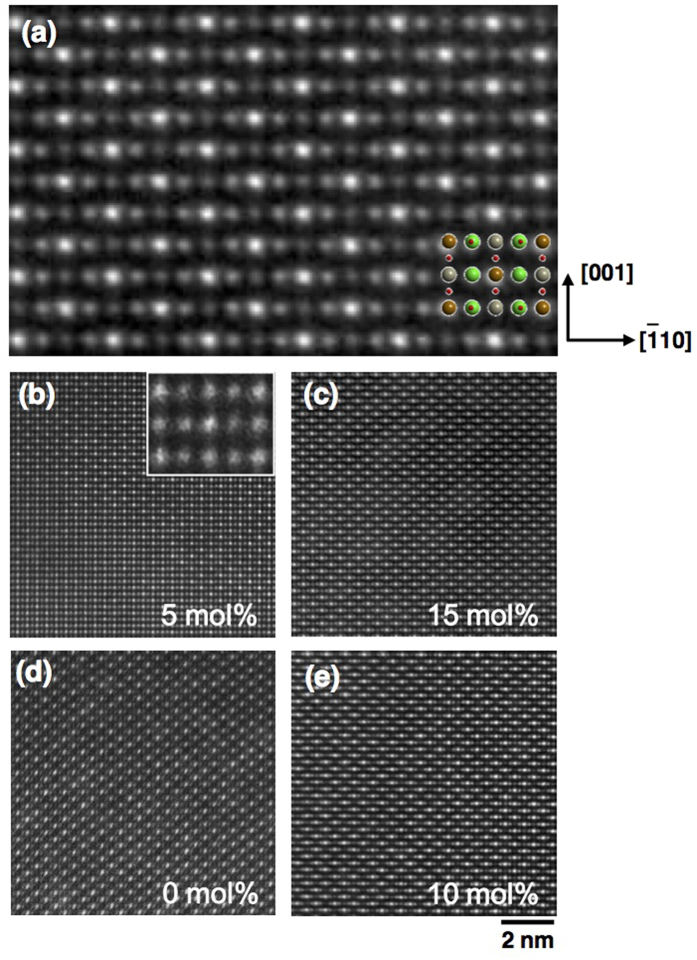
HADDF-STEM images of SFRO-xRe and SCRO-xRe. (**a**) In the 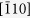 projection, Sr (green), Fe (gray), Re (brown), and O (red) atoms are well distinguished by *Z* contrast. This is a reference image taken from the most cationic ordered SFRO-15Re. The images of (**b**) SFRO-5Re, (**c**) SFRO-15Re, (**d**) SCRO-0Re, and (**e**) SCRO-10Re are displayed. The quantities of AS defects are noted in the bottom right corner of each figure. The inset of (**b**) is a magnified AS defect region indicated by the yellow rectangle, which shows a clear difference with well-ordered structure of (**a**).

We then systematically look at microscopic structure of AS defect in SFRO and SCRO using HAADF-STEM by comparing *Z* contrasts. Sequential arrangements of Sr-Fe-Sr-Re-Sr atoms are clearly imaged in the [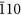] projection, since B-site atoms are arranged along the <110> direction (Fig. 2a). The effect of excess Re on the AS configurations is understood by the series of images from SFRO-5Re, SFRO-15Re, SCRO-0Re, and SCRO-10Re (Fig. 2b–e). In SFRO-5Re, defect clusters are clearly imaged in a form of antiphase-boundary-like structure, similar to the findings for Sr_2_FeMoO_6_^7^, and pure SFRO^12^. They are not seen in SFRO-15Re since the size of clustered AS defects decreases with an increase of excess Re. However, for both SCRO-0Re and SCRO-10Re, no defective structures are found. The level of cationic ordering in SCRO-0Re still appears to be high, even with an AS defect concentration of 16.446%. It is supposed that considering the limitation of the short-range imaging technique of HAADF-STEM, AS defects are scattered in the whole samples, as in other oxides^23^,^24^.

**Figure 3:**
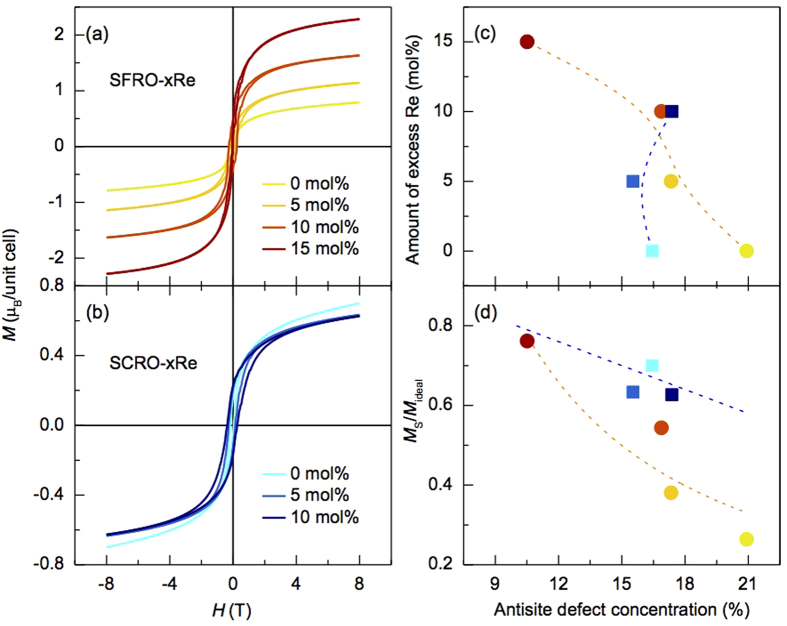
Magnetization (*M*)-magnetic field (*H*) hysteresis loops measured at 300 K for (**a**) SFRO-xRe and (**b**) SCRO-xRe. (**c**) The amount of excess Re and (**d**) the normalized saturated-magnetizations (*M*_S_/*M*_ideal_) of SFRO-xRe and SCRO-xRe are plotted for different AS defect concentrations. The dotted lines are a guide for visualization. The blue dotted line in (**d**) corresponds to the formula, *M*_*S*_/*M*_ideal_ = (1 − 2*x*) (*m*_*Fe*_ − *m*_*Re*_)/3 μ_B_ or *M*_*S*_/*M*_ideal_ = (1 − 2*x*) (*m*_*Cr*_ − *m*_*Re*_)/1 μ_B_. In (**c,d**), the colors of the closed circles and squares correspond to those of lines in (**a,b**), respectively.

The magnetic property is examined via magnetic hysteresis-loop measurement, (Fig. 3). Since XRD and HAADF-STEM experiments had been performed at room temperature, *M*_S_ was also measured at 300 K for SFRO and SCRO samples. *M*_S_ increases dramatically for SFRO while it decreases monotonously for SCRO with an increase of excess Re amount (see Table 1 and Fig. 3). Figure 3d shows the evaluated ratio of *M*_S_ to the ideal magnetic moment *M*_ideal_ (*M*_S_/*M*_ideal_) in terms of the defect concentration. *M*_ideal_ is 3 μ_B_/unit cell in SFRO and 1 μ_B_/unit cell in SCRO, assumed by the antiferromagnetic coupling of Fe^3+^ - Re^5+^ and Cr^3+^ - Re^5+^. *M*_S_/*M*_ideal_ reaches 0.76 for SFRO and 0.63 for SCRO with a minimum concentration of AS defects, and it changes as the concentration increases.

We can simply predict the magnetic saturation as a function of cationic ordering using the proposed formula assuming a spin-only contribution^5^. In our cases, two equations can be constructed as follows,


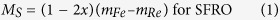



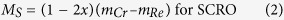


where *m*_*Fe*_, *m*_*Cr*_, and *m*_*Re*_ are the magnetic moments of Fe, Cr, and Re ions, respectively; *x* is the percentage of AS defects. However, the equations do not work well. For example, one SFRO sample, containing a defect concentration of 20.926%, exhibits *M*_S_ of ~0.79 μ_B_/unit cell, which is much lower than the predicted *M*_S_ of ~1.74 μ_B_/unit cell. In contrast, one of SCRO samples having a concentration of 17.378% possesses *M*_S_ of ~0.62 μ_B_/unit cell, similar to the predicted value of ~0.65 μ_B_/unit cell. This implies that the magnetic saturation is affected by other factors besides the defect concentration. It is natural to consider Re vacancies as the factor since Re vacancies are known to be likely to form in the oxides. We thus estimate the formation energies of Re vacancies using first-principles calculations. Formation energy is the energy required to form a defect in the host material. The calculated formation energies are higher than 3.5 eV, indicating that the vacancies formation can be successfully suppressed via adding excess Re (Table S2). Therefore, Re vacancies negligibly contribute to the magnetic properties of SFRO and SCRO with excessive Re.

We propose that both concentration and configuration of AS defects are detrimental to the magnetic properties, based on the followings: (i) *M*_S_/*M*_ideal_-dependence on the concentration of AS defects in SFRO quite differs from that in SCRO; i.e. *M*_S_/*M*_ideal_ for SFRO is much more sensitive to the defect concentration. *M*_S_/*M*_ideal_ reaches only ~0.38 for SFRO-5Re and ~0.62 for SCRO-10Re, although the samples contain similar concentrations of AS defects, 17.378% in SCRO and 17.340% in SFRO (Fig. 3d and Table 1). (ii) The defect configurations are totally different. The AS defects form an antiphase boundary in SFRO-5Re (Fig. 2b) but are scattered in SCRO-10Re (Fig. 2d). These observations indicate that AS defect clustering significantly impact on the magnetization, which can be explained in terms of the pinning effect on the magnetic domains, as in an exchange bias system. When antiferromagnetic and ferro- or ferrimagnetic domains coexist, i.e., ferrimagnetic and antiferromagnetic spins from ordered and disordered cationic atoms in the oxides, respectively, the interfacial antiferromagnetic spins can be pinned or unpinned according to the size-dependent anisotropy energy of antiferromagnetic domains^25^. As the size of the antiferromagnetic domains increases, the anisotropy energy also increases and the interfacial spins are more likely to be pinned; i.e., they hardly rotate in the external magnetic field directions and remaining *M*_S_.

**Figure 4:**
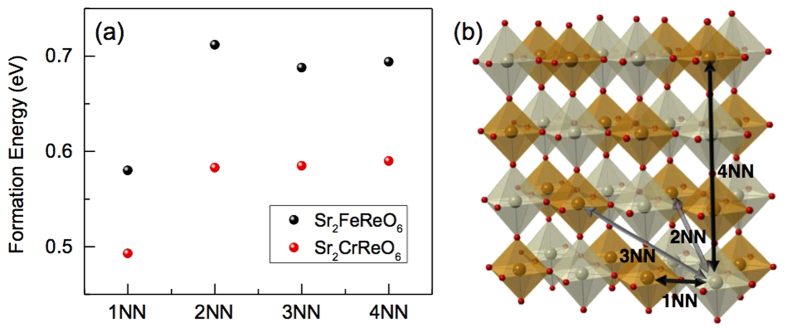
(**a**) Formation energies of Fe_Re_-Re_Fe_ for SFRO (black spheres) and Cr_Re_-Re_Cr_ for SCRO (red spheres) are plotted as a function of interatomic distance. (**b**) The detailed interatomic distance is depicted in SFRO unit cells. FeO_6_ and ReO_6_ octahedra are sketched with brown and gray colors, respectively.

To support, the formation energies and spin moments for isolated AS defects, i.e., Fe_Re_ and Re_Fe_ in SFRO and Cr_Re_ and Re_Cr_ in SCRO, and their clusters are theoretically investigated with various configurations. Overall, easy formation with the opposite spin moment of the defects is consistent with our experimental observations of lowered net magnetization with high concentrations of AS defects. All the isolated configurations have low formation energies of nearly less than 1eV, and hence they are likely to form as dominant defects in the oxides (Table S2). Thus, the concentration of AS defects would be high, and interactions between the defects can occur and form clusters. As shown in Fig. 4, two species of isolated AS defects, Fe_Re_ and Re_Fe_ in SFRO energetically favor to closely locate and form a cluster, Fe_Re_-Re_Fe_ (1NN) with a low formation energy of ~0.6 eV. Similarly, the Cr_Re_-Re_Cr_ pair (1NN) is the most stable with a formation energy of ~0.5 eV. Another cluster configuration, composed of the same species of an AS defect, is also examined. Our calculations show that the binding energy is 0.11 eV for Fe_Re_-Fe_Re_ and 0.03 eV for Re_Fe_-Re_Fe_ in SFRO, and is 0.13 eV for Cr_Re_-Cr_Re_ and 0.02 eV for Re_Cr_-Re_Cr_ in SCRO. All the values are positive, indicating that forming such a cluster is endothermic. Regarding the magnetic feature, the direction of spin-magnetic moment of isolated AS defects is opposite to that of the sublattice where they are positioned. The absolute values for Fe_Re_ (−4.13 μ_B_) and Cr_Re_ (−2.85 μ_B_) are comparable to those for the host atoms, and those for Re_Fe_ (2.21 μ_B_) and Re_Cr_ (2.18 μ_B_) are larger than those of the host Re atom (Table S1). Likewise, the magnetic moments of the participated AS defects in the 1NN configuration are calculated to be nearly same to those of each isolated AS configuration.

**Figure 5:**
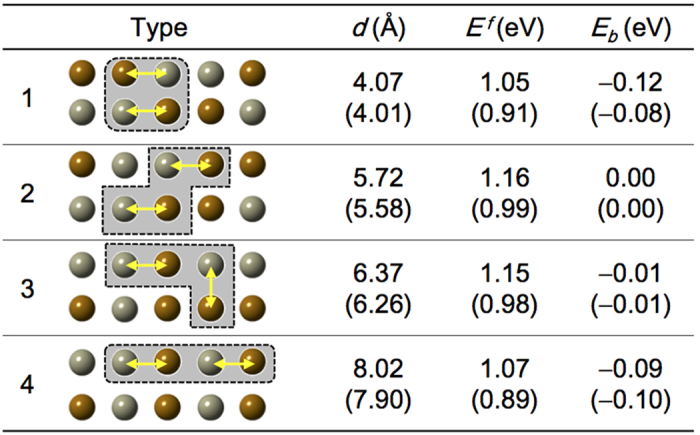
The interatomic distance (*d*), formation energy (*E*^*f*^), and binding energy (*E*_*b*_) for four different atomic configurations of several clusters composed of two 1NN pairs in SFRO. The values for SCRO are shown in parentheses. Brown (gray) spheres and yellow arrows indicate Re (Fe) atoms and single Fe_Re_-Re_Fe_ pair in SFRO, respectively.

Finally, in order to address the stability of an antiphase-boundary-like feature in SFRO, the interactions between two 1NN pairs are investigated by considering four neighboring configurations, i.e., type 1 to 4, as shown in Fig. 5, where type 1 has a strong tendency to cluster AS defects. In SFRO, type 1 has the lowest formation energy as well as the strongest binding energy among the considered configurations; hence two pairs may lie close to each other. In contrast, the type 4 configuration is the most stable in SCRO, where two pairs locate apart. This may give an explanation to the experimental observation that an antiphase-boundary-like feature exists in the SFRO samples, but not in the SCRO samples.

## Conclusions

We have investigated cationic ordering and its impact on the magnetic property in SFRO and SCRO through a series of experiments and computations. (i) With an increase of extra Re, the concentration of AS defects decreases in the SFRO samples but slightly increases in the SCRO samples. (ii) Excess Re enhances ferrimagnetic feature of SFRO but lowers the net magnetization of SCRO. (iii) When both oxides contain AS defects with similar concentrations, AS defects are clustered in an antiphase-boundary-like microstructure in SFRO due to attractive interaction between them, whereas they are spatially distributed in the whole SCRO samples. (iv) The cluster-type AS defects in SFRO deteriorated the net magnetization much more drastically compared to the scattered case in SCRO. Herein, we raise the issue of AS defect configuration in terms of whether the defects are clustered or scattered, and show that the magnetization is strongly dependent on the defect configuration as well as the amount of defects.

## Additional Information

**How to cite this article**: Lim, T.-W. *et al*. Insights into cationic ordering in Re-based double perovskite oxides. *Sci. Rep.* **6**, 19746; doi: 10.1038/srep19746 (2016).

## Supplementary Material

Supplementary Information

## Figures and Tables

**Table 1:** The measured XRD intensity ratio of (110) and (011) peaks and the calculated AS defect concentrations are displayed for SFRO-xRe and SCRO-xRe.

**Amount of excess Re**	***I***_**110**_**/*****I***_**011**_	**AS defect concentration (%)**
SFRO		
0 mol%	0.8193	20.926
5 mol%	0.6840	17.340
10 mol%	0.4937	16.878
15 mol%	0.4618	10.512
SCRO		
0 mol%	0.5011	16.446
5 mol%	0.4989	15.528
10 mol%	0.6294	17.378
