# Supplementary material for: Insights into cationic ordering in Re-based double perovskite oxides
Source: Sci Rep. 2016 Jan 25;6:19746. doi: 10.1038/srep19746 (PMC4726128; doi:10.1038/srep19746)
Supplement: Supplementary Information [file srep19746-s1.doc]

**Supplementary Materials**

**Insights into cationic ordering in Re-based double perovskite oxides**

Tae-Won Lim,^†,‡^ Sung-Dae Kim,^‡^ Kil-Dong Sung,^‡^ Young-Mok Rhyim,^‡^ HyungJeen Jeen,^¶^ Jondo Yun,^†^ Kwang-Ho Kim,*^#^* Ki-Myung Song,^§^ Seongsu Lee,^§^ Sung-Yoon Chung, ^§§^ Minseok Choi,^*,‡^ and Si-Young Choi^*,‡^

**Methodology**

**Formation energy calculations**

The formation energy of a defect *D* is defined as:^[1,2]^

$$E^{f}(D)=E_{\text{tot}} (D)-E_{\text{tot}}(\text{Oxide})-\sum_{i} n_{i}\mu_{i}\text{,}$$

where *E*_tot_(*D*) is the total energy of a supercell containing a defect *D*, and *E*_tot_(Oxide) is the total energy of perfect SFRO or SCRO supercell. *n_i_* is the number of atoms of type *i* added to (*n_i_* > 0) and/or removed from (*n_i_* <0) the perfect crystal to form the defect, and *μ_i_* (*i* = Sr, Fe, Cr, Re, and O) are the atomic chemical potentials. In this formalism the chemical potentials *μ_i_* are variable, yet restricted by the formation of limiting phases containing Sr, Fe, Cr, Re, and O. The chemical potentials *μ_i_* must satisfy the stability condition of SFRO and SCRO:

$$2\mu_{\text{Sr}}+\mu_{\text{Fe}}+\mu_{\text{Re}}+6\mu_{\text{O}}=\Delta H_{f}(\text{SFRO}),$$

$$2\mu_{\text{Sr}}+\mu_{\text{Cr}}+\mu_{\text{Re}}+6\mu_{\text{O}}=\Delta H_{f}(\text{SCRO}),$$

with *μ*_Sr_ ≤ 0, *μ*_Fe_ ≤ 0, *μ*_Cr_ ≤ 0, *μ*_Re_ ≤ 0, and *μ*_O_ ≤ 0. *△H_f_* (SFRO) and *△H_f_* (SCRO) are the formation enthalpy of perfect SFRO and SCRO crystals, respectively. The chemical potentials *μ*_Sr_, *μ*_Fe_, *μ*_Re_, and *μ*_O_ for SFRO are further constrained by the formation of SrO, Fe_2_O_3_, ReO_3_, Sr_3_(FeO_3_)_2_, and Sr_5_(ReO_6_)_2_ phases:

$$\mu_{\text{Sr}}+\mu_{\text{O}}\leq\Delta H_{f}(\text{SrO})\text{,}$$

where *△H_f_* (Oxide) is the formation enthalpy of each the oxide. In the same sense, SrO, Cr_2_O_3_, ReO_3_, Sr_2_CrO_4_, and Sr_5_(ReO_6_)_2_ are considered for SCRO.

SFRO and SCRO films or powders are usually grown using techniques such as solid-state reaction under Re-excess growth condition since Re excess helps to obtain the stoichiometric samples.^[3]^ As an estimate for the conditions during growth we set the Re chemical potential to *μ*_Re_ = 0 eV, corresponding to Re-rich limit. Figure S1 shows the phase diagram of SFRO and SCRO based on the calculated atomic chemical potentials.


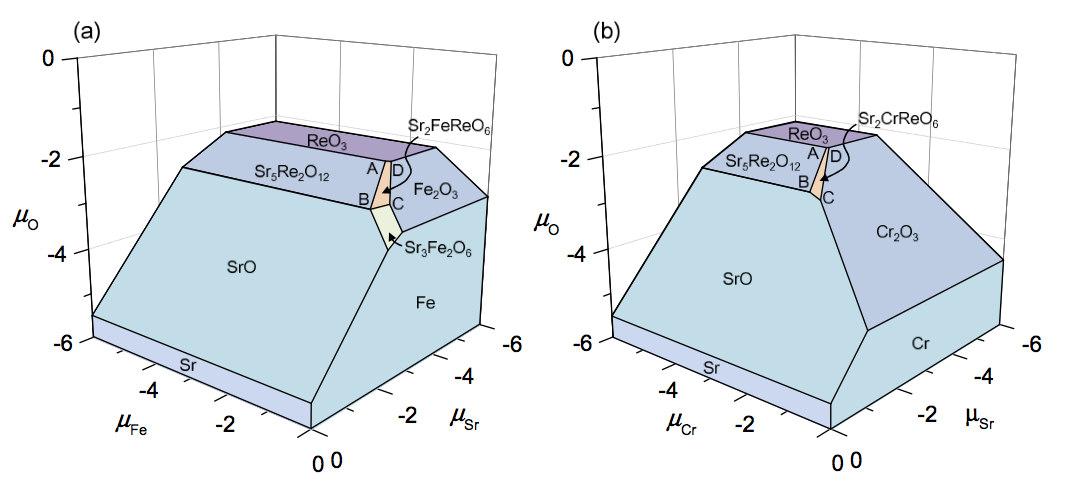


Figure S1. Allowed values of atomic chemical potentials defining the stability of a) SFRO and b) SCRO. The chemical potentials *μ*_Sr_, *μ*_Fe_, *μ*_Cr_, *μ*_Re_, and *μ*_O_ are limited by the formation of various secondary phases. *μ*_Re_ is set to zero to represent defect energetics in the oxides grown under a Re-excess environment.

Table S1. Lattice parameters and spin-magnetic moment of SFRO and SCRO from GGA+*U* and experiment. The values in parenthesis are from HSE06. Negative sign of Re magnetic moment indicates an opposite direction of magnetic moments of Fe and Cr atoms.

| Property | Sr_2_FeReO_6_ | | | |
| --- | --- | --- | --- | --- |
|  | *a* (Å) | *c* (Å) | *m*_Fe_ (μ_B_) | *m*_Re_ (μ_B_) |
| Present | 5.615 | 8.024 | 4.12 (4.15) | -1.46 (-1.43) |
| Experiment | 5.561^[4]^ | 7.901^[4]^ | 4.35^[5]^, 3.6^[6]^ | -0.16^[5]^, -1.0^[6]^ |
|  |  |  |  |  |
| Property | Sr_2_CrReO_6_ | | | |
|  | *a* (Å) | *c* (Å) | *m*_Cr_ (μ_B_) | *m*_Re_ (μ_B_) |
| Present | 5.588 | 7.960 | 2.74 (2.72) | -1.72 (-1.55) |
| Experiment | 5.527^[4]^ | 7.809^[4]^ | 2.52^[5]^ | -0.21 |

Table S2. Formation energy of isolated AS defects in SFRO and SCRO in eV unit. The growth conditions (A–D) are taken from Figure S1. For comparison, the formation energy of Re vacancies is also included.

| Condition | Defect formation energy in SFRO (eV) | | | |
| --- | --- | --- | --- | --- |
|  | A | B | C | D |
| Fe_Re_ | 0.69 | 0.04 | -0.20 | 0.58 |
| Re_Fe_ | 0.01 | 0.66 | 0.90 | 0.12 |
| V_Re_ | 4.00 | 4.00 | 4.00 | 4.00 |
|  |  |  |  |  |
| Condition | Defect formation energy in SCRO (eV) | | | |
|  | A | B | C | D |
| Cr_Re_ | 0.59 | -0.12 | -0.49 | 0.54 |
| Re_Cr_ | 0.01 | 0.72 | 1.09 | 0.06 |
| V_Re_ | 3.54 | 3.54 | 3.54 | 3.54 |

**References**

[1] C. G. Van de Walle, J. Neugebauer, *J. Appl. Phys*. **2004**, *95*, 3851–3879.

[2] F. Oba, M. Choi, A. Togo, I. Tanaka, *Sci. Technol. Adv. Mater.* **2011**, *12*, 034302.

[3] J. B. Lim, S.-Y. Choi, M.-H. Kim, D. Suvorov, J.-H. Jeon, T.-K. Song, W.-J. Kim, *Mater. Lett.* **2012**, *75*, 143-145.

[4] H. Kato, T. Okuda, Y. Okimoto, Y. Tomioka, K. Oikawa, T. Kamiyama, Y. Tokura, Y. *Phys. Rev. B* **2004**, *69*, 184412.

[5] J. M. De Teresa, D. Serrate, C. Ritter, J. Blasco, M. R. Ibarra, L. Morellon, W. Tokarz, *Phys. Rev. B* **2005**, *71*, 092408.

[6] M. Retuerto, M. Martinez-Lope, M. Garcia-Hernandez, J. Alonso, *Mater. Res. Bull.* **2009**, *44*, 1261 – 1264.
